# Clinical research review: usefulness of bovine lactoferrin in child health

**DOI:** 10.1007/s10534-022-00430-4

**Published:** 2022-08-09

**Authors:** Momoko Miyakawa, Hirotsugu Oda, Miyuki Tanaka

**Affiliations:** Food Ingredients and Technology Institute, R&D Division, Morinaga Milk Industry, Co., Ltd., Zama, Kanagawa Japan

**Keywords:** Lactoferrin, Clinical study, Child, Infant, Review

## Abstract

Lactoferrin (LF) is abundant in human milk and plays an important role in the health of children. Bovine LF (bLF) has high homology with human LF and has been reported to have multiple biological functions. Several clinical studies have been conducted considering these properties, which reported the usefulness of bLF. This review was aimed to provide an overview of the clinical evidence in children. We searched clinical reports investigating the effects of bLF in children and identified 36 studies on the role of bLF in infections, iron metabolism, body growth, cerebral development, and fecal microbiome. Considering the accumulated evidence, bLF may contribute to the child health, particularly by suppressing or alleviating gastrointestinal and respiratory symptoms, and improving the iron status of children with anemia or those at high risk of anemia. The dose of bLF varies depending on the expected effect and target age, but may not necessarily have to be as high as human LF in human milk. Some of the beneficial effects of bLF have not been fully validated due to limited clinical evidence or being observed in the secondary analysis of some studies. Further clinical evidence would add significant value to the use of bLF in child health.

## Introduction

Lactoferrin (LF) is an iron-binding glycoprotein and well-known for its multiple biological functions such as antimicrobial and immunomodulatory activities. LF is abundant in human breast milk and its concentration is maximum during early lactation, which rapidly declines thereafter and remains relatively constant from one month to two years of lactation (Rai et al. 2014). This means that LF is an important component for infants and children even after the age of one year.

Human and bovine LF (hLF and bLF, respectively) share 69% amino acid sequence identity based on the protein sequence alignment analysis (Pierce et al. 1991), and their bioactivities as assessed in vitro or in animal models are comparable (Steijns and van Hooijdonk 2000; Lönnerdal et al. 2011). bLF is manufactured on an industrial scale and used worldwide in a variety of food products such as powdered formula. From the 1980s to the present, multiple clinical studies have been conducted in children fed bLF. In this review, we focus on clinical studies that examined the efficacy of bLF in children and provide an overview of the potential contribution of bLF intake to child health.

## Materials and methods

We searched clinical reports investigating the effects of bLF in children using PubMed, Google Scholar, and additional hand searching. In this review, we identified 36 studies and obtained full-length papers. The main effects discussed in these studies were protection from infection, modulation of iron metabolism, body growth, cerebral development, and regulation of the fecal microbiome.

## Results and discussion

### Suppressive and alleviative effects on symptoms of infections

We identified 10 published clinical studies that investigated the effects of bLF on the symptoms of infections (Table 1).

**Table 1:** Clinical studies on the effects of bLF on symptoms of infections in children

Refs., study design	Population, country	bLF intake, form	Control	Intervention period	Results
Okuda et al. (2005) RDBPCT	59 *H. pylori*-infected healthy subjects, 3–59 years (Japan)	400 mg, tablet	Placebo tablet	12 weeks	Higher number of subjects with 50% decrease in the UBT value at 12 weeks
King et al. (2007) RDBPCT	79 healthy, formula-fed, near-term infants, < 4 weeks (USA)	850 mg/L, formula	Commercial formula; bLF 102 mg/L	~ 12 months of age	Fewer episode of lower respiratory tract illness, primarily wheezing. No difference in the frequency of diarrhea, upper respiratory illness, otitis media, and other illnesses
Ochoa et al. (2008) RDBPCT	52 weaned children, 12–36 months (Peru)	1 g	Maltodextrin	9 months	No difference in overall incidence and prevalence rates of diarrhea. Lower prevalence of colonization with *Giardia*
Yen et al. (2011) RSBT	216 healthy children, 2–6 years (Taiwan)	70–85 mg, formula	bLF-free formula	–	No difference in the incidence or severity of enterovirus or rotavirus infection
Ochoa et al. (2013) RDBPCT	555 weaned children, 12–18 months (Peru)	1 g	Maltodextrin	6 months	No difference in diarrhea incidence rate. Lower longitudinal prevalence and duration of diarrhea and episode with dehydration and total loose stool
Chen et al. (2016) RDBPCT	260 exclusively-breastfed before the study, healthy, term infants, 4–6 months (China)	35.8 mg, formula	bLF-free formula	3 months	Lower morbidity and duration of respiratory- and diarrhea-related illness. Fewer symptoms of rhinorrhea, cough, and wheezing
Björmsjö et al. (2020) RDBCT	180 healthy, formula-fed, full-term infants, 6 ± 2 weeks (Sweden)	1 g/L, formula	bLF-free formula	~ 6 months of age	No benefit on gastrointestinal symptoms
Motoki et al. (2020) RDBPCT	109 healthy children, 12–32 months (Japan)	48 mg, formula	bLF-free formula	13 weeks	Reduced prevalence of acute gastrointestinal symptoms and total number of days of respiratory symptoms
Tsukahara et al. (2020) ROT	1296 children in nursery schools, 3–6 years (Japan)	100 mg, yogurt	Non-ingestion	15 weeks	No differences in the numbers of nursery school absentees or absent days. Reduced number of absentees due to vomiting and absent days due to illnesses in children consuming bLF yogurt ≥ 3 days/week
Chen et al. (2021) RCOT	105 infants, exclusively-breastfed before the study, with anemia, 6–9 months (China)	47.2 mg, 91.5 mg, formula	bLF-free formula	3 months	Lower morbidity of rhinorrhea, wheezing, skin rash, vomiting, and nausea in the bLF 47.2 group. Lower morbidity of respiratory-related illness, wheezing, diarrhea-related illness, diarrhea, vomiting, and nausea in the bLF 91.5 group. Higher bLF dose was more effective in preventing diarrhea-related illness, diarrhea, vomiting, and nausea

*RDBPCT* randomized, double-blind, placebo-controlled trial; *H. pylori Helicobacter pylori*; *UBT* urea breath test; *RSBT* randomized, single-blind trial; *RDBCT* randomized, double-blind, controlled trial; *ROT* randomized, open trial; *RCOT* randomized, controlled, open trial

Although the methods for evaluating the outcomes varied depending on the study, 8 studies (Okuda et al. 2005; King et al. 2007; Ochoa et al. 2008, 2013; Chen et al. 2016, 2021; Motoki et al. 2020; Tsukahara et al. 2020) reported beneficial effects of bLF intake in children. Among them, seven studies (Okuda et al. 2005; Ochoa et al. 2008, 2013; Chen et al. 2016, 2021; Motoki et al. 2020; Tsukahara et al. 2020) showed suppressive or alleviative effects on gastrointestinal symptoms, and four studies (King et al. 2007; Chen et al. 2016, 2021; Motoki et al. 2020) showed such effects on respiratory symptoms.

A meta-analysis of clinical studies on adults suggested that bLF could increase the eradication rate and suppress the side effects of anti-*H. pylori* therapy (Zou et al. 2009). On the other hand, clinical studies on the effect of bLF on *H. pylori* infection in children are limited and have not yet been fully investigated (Okuda et al. 2005). As an overall trend, numerous clinical studies on *H. pylori* eradication by bLF were conducted in the early 2000s, but only a few studies have been conducted in recent years, probably owing to the improvement in drinking water and standard anti-*H. pylori* therapy.

A recent meta-analysis including studies on adults and children suggested that bLF could reduce the risk of respiratory tract infections (Ali et al. 2021) supporting the usefulness of bLF in the management of respiratory infections in children.

Based on basic research, the mechanisms underlying these protective effects of bLF can be attributed to its antibacterial, antiviral, immunomodulatory activities, and barrier functions.

bLF exhibits antibacterial activity against various bacteria mainly by sequestering iron, which is essential for bacterial growth (Arnold et al. 1977), or by directly binding with lipopolysaccharide (LPS) embedded in the gram-negative bacterial surface, leading to destabilization and permeability of the bacterial outer membrane (Drago-Serrano et al. 2012). bLF and lactoferricin, an antimicrobial peptide derived from pepsin digestion of bLF, have been demonstrated to exhibit antibacterial activity against *H. pylori *in vitro (Dial et al. 1998).

Antiviral properties of bLF have also been extensively studied (Oda et al. 2020a). In vitro studies have suggested that bLF exerts antiviral activity against viruses that infect the gastrointestinal tract through inhibition of viral attachment to their target cells and suppression of viral replication via interferons (IFNs) (Superti et al. 1997; Ishikawa et al. 2013; Shin et al. 2017; Oda et al. 2021).

bLF also exerts immunomodulatory effects. Intelectin, a receptor for LF, is expressed in the small intestine, particularly the epithelium overlying the Peyer’s patches (Talukder et al. 2003) where immune cells are located, suggesting that it may mediate the interaction between bLF and immune cells. In particular, orally-administered bLF activates natural killer (NK) cells via type I IFN (Kuhara et al. 2006). Moreover, bLF activates CD4^+^ and CD8^+^ T cells and enhances the production of IgA from plasma cells (Arciniega-Martínez et al. 2016). Recently, bLF has been reported to activate human plasmacytoid dendritic cells in adults, which produce type I IFN, activate NK cells, CD4^+^ and CD8^+^ T cells, and B cells, and play an important role in systemic viral clearance by modulating innate and adaptive immune responses (Miyakawa et al. 2021a). In contrast, bLF suppresses the production of inflammatory cytokines such as interleukin (IL)-6 and tumor necrosis factor (TNF)-α (Zimecki et al. 1999).

bLF induces the growth and differentiation of enterocytes, strengthens the tight junctions, and thus enhances the integrity of intestinal epithelia (Zhao et al. 2019). bLF also induces the production of type I and type III IFNs, antiviral cytokines from enterocytes (Shin et al. 2017; Miyakawa et al. 2021b).

Collectively, orally-administered bLF exerts local antibacterial and antiviral activities, local and systemic immunomodulatory activity, and strengthens the intestinal barrier, leading to suppression and alleviation of gastrointestinal and respiratory symptoms.

The dose of bLF varied in different studies, and ranged from 35.8 to 1000 mg per day. Some studies were conducted using a high dose of bLF, which is almost equivalent to the amount of hLF that infants could consume from breast milk; on the other hand, some studies were conducted using a relatively low dose of bLF as that contained in commercially available formula. In a study, dose-dependent efficacy was observed with 91.5 mg of bLF being more effective than 47.2 mg of bLF in suppressing gastrointestinal symptoms (Chen et al. 2021). While, 200 mg of bLF has been reported to prevent acute gastrointestinal and respiratory symptoms (Mizuki et al. 2020; Miyakawa et al. 2021b) and shorten the duration of infectious diseases (Oda et al. 2020b) in adults. Considering body weight, the dose of bLF might not necessarily have to be as high as that found in human breast milk to exert beneficial effects in children.

Two studies (Yen et al. 2011; Björmsjö et al. 2020) showed no beneficial effects of bLF fortification; subject characteristics, public health environment, or disease burden might have affected these results. The quality of bLF such as iron saturation, contaminant, and degradation might vary depending on the suppliers, and it might also affect the results. Properties of bLF were compared among suppliers by several in vitro assays, but no consistent features were observed (Lönnerdal et al. 2021). It is desirable to develop good in vitro assay models for each biological functions to be evaluated in human, and assess the activities of bLF prior to conducting clinical studies.

Five studies (Egashira et al. 2007; Zuccotti et al. 2009; Tolone et al. 2012; Cheng et al. 2019; Li et al. 2019) showed the beneficial effects of bLF in combination with other components often contained in breast milk or formula such as bifidobacteria, probiotics, lactulose, lysozyme, or milk fat globule membrane (MFGM), although relative contribution of each component was not determined (Table 2). Combination with other functional components may produce a synergistic positive effect on the child health.

**Table 2:** Clinical studies on the effects of bLF with other components on symptoms of infections in children

Refs., study design	Population, country	bLF intake, form	Control	Intervention period	Results
Egashira et al. (2007) Open study	298 children, 0–4 years (Japan)	100 mg, + bifidobacteria/lactulose, supplement or yogurt	Non-ingestion	12 weeks	No difference in the incidence of rotavirus gastroenteritis. Decreased duration and frequency of diarrhea and vomiting
Zuccotti et al. (2009) Before-after study	10 children with RRTI, 3–7 years (Italy)	2.7 g, + curcumin	–	4 weeks	Reduction in the number of respiratory tract infections in 8/10 cases during one year follow up. Skewed CD8^+^ T cell maturation. Increased CD14^+^/TLR2^+^ cells and decreased CD14^+^/TLR4^+^ cells
Tolone et al. (2012) ROT	68 *H. pylori*-infected children, 8.3 ± 3.4 years (Italy)	Unknown, + probiotics/standard treatment	Standard treatment	7 days	Higher success rate of *H. pylori* eradication, but the difference was not significant
Cheng et al. (2019) RDBPCT	235 children, 12–23 months (Malawi)	1.5 g + lysozyme, rice powder	bLF-free, lysozyme-free rice powder	16 weeks	Improved gut function. Lower rates of hospitalization and development of acute malnutrition
Li et al. (2019) RDBPCT	451 healthy, formula-fed, term infants, 10–14 days (China)	0.6 g/L, + MFGM, formula	bLF-free, MFGM-free formula	~ 365 days of age	Lower incidence of respiratory-associated events and diarrhea

*RRTI* recurrent respiratory tract infection; *TLR* toll-like receptor; *ROT* randomized, open trial; *H. pylori Helicobacter pylori*; *RDBPCT* randomized, double-blind, placebo-controlled trial; *MFGM* milk fat globule membrane

### Immunomodulatory effect

Some clinical studies have investigated the effect of bLF on immune responses in children and demonstrated its immunomodulatory effect to some extent (Table 3).

**Table 3:** Clinical studies on the effect of bLF on immunomodulation in children

Refs., study design	Population, country	bLF intake, form	Control	Intervention period	Results
Zuccotti et al. (2006) Before-after study	22 HIV-1 vertically infected children, 3–18 years (Italy)	3 g,	–	6 months	Reduction in plasma viral load and increase in the percentage of CD4^+^ cell count
Zuccotti et al. (2007) Before-after study	11 HIV-infected ARV-naive children, 4–17 years (Italy)	3 g	–	4 weeks	Skewed CD4^+^ and CD8^+^ T cell maturation. Increased phagocytosis and killing by CD13^+^ phagocytes. Increased CD14^+^/TLR2^+^ cells, and the IL-12/IL-10 ratio
Yen et al. (2011) RSBT	216 healthy children, 2–6 years (Taiwan)	70–85 mg, formula	bLF-free formula	–	No difference in IFN-γ or IL-10 serum levels
Chen et al. (2021) RCOT	105 infants, exclusively-breastfed before the study, with anemia, 6–9 months (China)	47.2 mg, 91.5 mg, formula	bLF-free formula	3 months	Higher HBD-2, LL-37, sIgA, and butyrate levels in the bLF 47.2 and bLF 91.5 groups. Lower calprotectin levels in the bLF 47.2 and bLF 91.5 groups

*HIV-1* human immunodeficiency virus type 1; *ARV* antiretroviral; *TLR* toll-like receptor; *IL* interleukin; *RSBT* randomized, single-blind trial; *IFN* interferon; *RCOT* randomized, controlled, open trial; *HBD* human beta defensing; *sIgA* secretory IgA

Modulation of T cells, phagocytes, monocytes, and Th1/Th2 balance has been observed in HIV-infected children after bLF intake (Zuccotti et al. 2006, 2007). However, it is uncertain whether these effects can be observed in healthy individuals. Combination of bLF with curcumin also modulated T cells and monocytes in children with recurrent respiratory tract infection (RRTI) (Zuccotti et al. 2009) (Table 2). Chen et al. observed dose-dependent effects of bLF on fecal biochemical indices such as human beta defensin 2 (HBD-2), cathelicidin LL-37, secretory IgA (sIgA), butyrate, and calprotectin in children (Chen et al. 2021).

These immunomodulatory actions could be a mechanism underlying the suppression of diarrhea and the regulation of the microbiome. Nonetheless, there are some limitations, such as few studies, small sample size, study design, and subject characteristics, including children with illness; therefore, additional clinical studies are needed to clarify whether bLF modulates the immune responses in children.

### Improvement in iron metabolism

We identified 16 published clinical studies that investigated the effect of bLF on iron metabolism (Table 4).

**Table 4:** Clinical studies on the effect of bLF on iron metabolism in children

Refs., study design	Population, country	bLF intake, form	Control	Intervention period	Results
Fairweather-Tait et al. (1987) -	36 healthy, full-term infants, 7 days (England)	420 mg, ^58^Fe-labeled bLF	^58^FeCl_3_ + ascorbic acid	3 days	No difference in iron retention
Schulz-Lell et al. (1991) -	16 healthy, term infants, 3 weeks	1 g/L, formula	bLF-free formula	3 days × 5 period	No difference in iron retention
Chierici et al. (1992) -	51 healthy, formula-fed, full-term infants, at birth (Italy)	0.1 g/L, 1.0 g/L, formula	bLF-free formula	150 days	Higher serum ferritin on day 90 and 150 in infants receiving formula with 1.0 g/L of bLF
Lönnerdal and Hernell (1994) -	50 healthy, term infants, 6 ± 2 weeks (Sweden)	4 mg/L iron (1.4 mg as bLF and 2.6 mg as FeSO_4_)	4 mg/L iron, 4 mg/L iron + Se, 4 mg/L iron + Cu, 7 mg/L iron	~ 6 months of age	No difference in hematological indices (Hb, serum iron, and MCV) between the groups at 6 months
Hernell and Lönnerdal (2002) -	57 healthy, term infants, 4 ± 2 weeks (Sweden)	1.8 mg/L iron (1.3 mg as bLF and 0.5 mg as FeSO_4_)	1.6 mg/L iron, 2.2 mg/L iron + nucleotide, 4 mg/L iron	~ 6 months of age	No difference in hematological indices (Hb, serum iron, MCV, and ferritin) between the groups at 4 and 6 months
King et al. (2007) RDBPCT	79 healthy, formula-fed, near-term infants, < 4 weeks (USA)	850 mg/L, formula	Commercial formula; bLF 102 mg/L	~ 12 months of age	Higher Ht at 9 months. No difference in Ht, Hb, and MCV at 12 months
Chen et al. (2015a) RDBPCT	260 exclusively-breastfed before the study, healthy infants, 4–6 months (China)	35.8 mg, formula	bLF-free formula	3 months	Higher Hb, ferritin, TFR-F index, and TBIC values. Lower prevalence of anemia, ID, and IDA
El-Khawaga and Abdelmaksoud (2019) ROT	94 children with IDA, 6–12 years (Egypt)	200 mg	Iron	30 days	Increase in Hb, RBC, MCHC, serum ferritin, and serum iron
Björmsjö et al. (2020) RDBCT	180 healthy, formula-fed, full-term infants, 6 ± 2 weeks (Sweden)	1 g/L, formula	bLF-free formula	~ 6 months of age	No difference in the iron status (Hb, ferritin, MCV, and serum iron) at 4 and 6 months
Chen et al. (2020) RCOT	105 infants, exclusively-breastfed before the study, with anemia, 6–9 months (China)	47.2 mg, 91.5 mg, formula	bLF-free formula	3 months	No remarkable difference in the bLF 47.2 group. Higher Hb level in infants of the bLF 91.5 group than those in the other two groups at 3 months of intervention
Mikulic et al. (2020) RSBCT	26 infants without severe anemia, 3–6 months (Kenya)	1.41 g apo-bLF + FeSO_4_, 1.41 g holo-bLF	FeSO_4_	28 days	Higher fractional iron absorption from the meal containing FeSO_4_ with apo-bLF than from the meal containing FeSO_4_ or holo-bLF
Atia et al. (2021) ROT	40 obese children with IDA, 6–18 year (Egypt)	100 mg	Ferric hydroxide polymaltose	3 months	Higher Hb, MCV, MCH, serum ferritin, serum iron, and transferrin saturation, and lower IL-6. No difference in hepcidin between the groups, but significant reduction was observed in the bLF group compared to that before intervention
El-Asheer et al. (2021) ROT	105 children with IDA, 2–15 years (Egypt)	100 mg ± iron sachet	Ferrous sulfate	30 days	Higher values of hematological parameters (RBC, Hb, Ht, MCV, MCH, serum iron, and reticulocyte) in the bLF/bLF + iron group compared with the iron group. Fewer adverse effects in the bLF group
El-Hawy et al. (2021) RCOT	120 children with IDA, 1–18 years (Egypt)	100–200 mg ± iron sachet	FeBC, IPC	1 month	Improved iron status in the FeBC and IPC groups compared with the bLF group, but no difference compared with the bLF + iron group. Higher side effects in the FeBC and IPC groups than in the bLF group
Kamal et al. (2021) -	150 children with IDA, > 2 years (Egypt)	bLF ± iron, dose unknown	Ferric hydroxide	3 months	Differences were observed in Hb, serum iron, and ferritin levels between the groups after 1.5 and 3 months of treatment, with the highest values in the bLF + iron group followed by the iron group
Omar et al. (2021) RCOT	70 children with CP and IDA, 1–10 years (Egypt)	200 mg sachet	IPC	4 weeks	Higher adjusted changes in Hb and serum ferritin and less frequent constipation

*Hb* hemoglobin; *MCV* mean corpuscular volume; *RDBPCT* randomized, double-blind, placebo-controlled trial; *Ht* hematocrit; *TFR*-F transferrin receptor-ferritin; *TBIC* total body iron content; *ID* iron deficiency; *IDA* iron deficiency anemia; *ROT* randomized, open trial; *RBC* red blood cell; *MCHC* mean corpuscular hemoglobin concentration,; *RDBCT* randomized, double-blind, controlled trial; *RCOT* randomized, controlled, open trial; *RSBCT* randomized, single-blind, crossover trial; *MCH* mean corpuscular hemoglobin; *IL* interleukin; *FeBC* iron bisglycinate chelate; *IPC* iron hydroxide polymaltose complex; *CP* cerebral palsy

Among the seven studies (Fairweather-Tait et al. 1987; Schulz-Lell et al. 1991; Chierici et al. 1992; Lönnerdal and Hernell 1994; Hernell and Lönnerdal 2002; King et al. 2007; Björmsjö et al. 2020) investigating the effect of bLF in healthy and near-term newborns, five (Fairweather-Tait et al. 1987; Schulz-Lell et al. 1991; Lönnerdal and Hernell 1994; Hernell and Lönnerdal 2002; Björmsjö et al. 2020) did not show any beneficial effect on iron absorption or iron status after bLF intake during the first six months. Healthy term infants with normal birth weight are born with iron stores sufficient for their growth during the first six months of life (Dallman et al. 1980). In some of the above studies (Lönnerdal and Hernell 1994; Hernell and Lönnerdal 2002; King et al. 2007; Björmsjö et al. 2020), after intervention, hematological parameters such as hemoglobin (Hb) or serum ferritin showed relatively high values in accordance with the WHO guidelines (World Health Organization 2001). Therefore, it was suggested that bLF was administered when iron demand was not high, and these conditions might have affected the results.

During the second half of infancy, the requirement for exogenous iron rapidly increases as the infant grows. In a study of infants who were previously exclusively breastfed and assumed to be at a high risk of anemia, after 3 months of bLF intervention, the iron status, including Hb, serum ferritin, transferrin receptor-ferritin (TFR-F) index, and total body iron content (TBIC) was improved, leading to a lower prevalence of anemia, iron deficiency (ID), and iron deficiency anemia (IDA) (Chen et al. 2015a). In addition, several studies on anemic children have shown that bLF consistently improved the iron status (El-Khawaga and Abdelmaksoud 2019; Chen et al. 2020; Mikulic et al. 2020; Kamal et al. 2021; El-Asheer et al. 2021; Omar et al. 2021; Atia et al. 2021). A recent meta-analysis of studies on pregnant women suggested that bLF improved IDA (Abu Hashim et al. 2017), and these findings are consistent with the results obtained from studies on children.

The mechanisms underlying the improvement in anemia by bLF are likely related to its anti-inflammatory activity. It has been reported that a part of anemia is associated with high levels of IL-6 that upregulates hepcidin, a peptide hormone mainly synthesized by hepatocytes. Hepcidin binds to ferroportin, causes its degradation, and leads to a significant decrease in iron export from the cells into plasma, and consequently, IDA may be detected (Rosa et al. 2017). Several studies on pregnant women (Paesano et al. 2009, 2010, 2012, 2014; Lepanto et al. 2018) suggest that bLF decreases IL-6 levels, restores iron delocalization, and improves hematological parameters. Atia et al. observed a decrease in serum IL-6 and hepcidin levels in anemic children after bLF ingestion (Atia et al. 2021), which suggests that bLF improves iron metabolism in children by a mechanism similar to that reported in adults. According to the secondary analysis of some studies, ingestion of bLF protected children from infections (Chen et al. 2016, 2021). This might be attributed to the decrease in inflammatory cytokines, including IL-6.

Furthermore, Mikulic et al. reported that apo-bLF might promote dietary iron absorption in anemic infants (Mikulic et al. 2020). bLF was reported to solubilize a > 70-fold molar equivalent of iron at neutral pH in vitro (Kawakami et al. 1993), suggesting that ingested bLF could solubilize iron in the intestine, promote iron absorption, and consequently, improve anemia. Furthermore, bLF may improve intestinal iron absorption in children by promoting the maturation of intestinal epithelia (Yang et al. 2014; Hu et al. 2019).

### Growth promotion

We identified 8 published clinical studies that reported the effects of bLF on anthropometric indices (Table 5).

**Table 5:** Clinical studies on the effect of bLF on body growth in children

Refs., study design	Population, country	bLF intake, form	Control	Intervention period	Results
Lönnerdal and Hernell (1994) -	50 healthy, term infants, 6 ± 2 weeks (Sweden)	4 mg/L iron (1.4 mg as bLF and 2.6 mg as FeSO_4_)	4 mg/L iron, 4 mg/L iron + Se, 4 mg/L iron + Cu, 7 mg/L iron	~ 6 months of age	No difference in weight and height
Hernell and Lönnerdal (2002) -	57 healthy, term infants, 4 ± 2 weeks (Sweden)	1.8 mg/L iron (1.3 mg as bLF and 0.5 mg as FeSO_4_)	1.6 mg/L iron, 2.2 mg/L iron + nucleotide, 4 mg/L iron	~ 6 months of age	Increased height and weight in the LF group compared with the nucleotide group
King et al. (2007) RDBPCT	79 healthy, formula-fed, near-term infants, < 4 weeks (USA)	850 mg/L, formula	Commercial formula; bLF 102 mg/L	~ 12 months of age	Trend of a greater increase in weight during the first six months
Ochoa et al. (2008) RDBPCT	52 weaned children, 12–36 months (Peru)	1 g	Maltodextrin	9 months	Higher height-for-age scores
Ochoa et al. (2013) RDBPCT	555 weaned children, 12–18 months (Peru)	1 g	Maltodextrin	6 months	Slightly lower height-for-age scores
Chen et al. (2015a) RDBPCT	260 exclusively-breastfed before the study, healthy infants, 4–6 months (China)	35.8 mg, formula	bLF-free formula	3 months	Higher weight, weight-by-age, and weight-by-height
Björmsjö et al. (2020) RDBCT	180 healthy, formula-fed, full-term infants, 6 ± 2 weeks (Sweden)	1 g/L, formula	bLF-free formula	~ 6 months of age	No difference in weight, length, and head circumference
Chen et al. (2020) RCOT	105 infants, exclusively-breastfed before the study, with anemia, 6–9 months(China)	47.2 mg, 91.5 mg, formula	bLF-free formula	3 months	No difference in weight, length, and head circumference

*RDBPCT* randomized, double-blind, placebo-controlled trial; *RDBCT* randomized, double-blind, controlled trial; *RCOT* randomized, controlled, open trial

Several studies have reported that bLF promotes body growth such as increase in height and weight (Hernell and Lönnerdal 2002; King et al. 2007; Ochoa et al. 2008; Chen et al. 2015a; Li et al. 2019).

We identified 3 published clinical studies that reported the effects of bLF with other components such as lysozyme and MFGM on anthropometric indices (Table 6). Two studies (Cheng et al. 2019; Li et al. 2019) showed the beneficial effects, although relative contribution of each component was not determined.

**Table 6:** Clinical studies on the effect of bLF with other components on body growth in children

Refs., study design	Population, country	bLF intake, form	Control	Intervention period	Results
Johnston et al. (2015) RDBPCT	480 healthy, formula-fed, term infants, 12–16 days (USA)	0.6 g/L, 1.0 g/L + PDX /GOS, formula	bLF-free, PDX -free, GOS-free formula	~ 365 days of age	No difference in growth rate
Cheng et al. (2019) RDBPCT	235 children, 12–23 months (Malawi)	1.5 g + lysozyme, rice powder	bLF-free, lysozyme-free rice powder	16 weeks	Greater increase in weight and mid-upper arm circumference in the control group
Li et al. (2019) RDBPCT	451 healthy, formula-fed, term infants, 10–14 days (China)	0.6 g/L, + MFGM, formula	bLF-free, MFGM-free formula	~ 365 days of age	Increased length on day 90, 180, and 275 and head circumference on day 30, 42, 60, 90, 180, and 275 in females

*RDBPCT* randomized, double-blind, placebo-controlled trial; *PDX* polydextrose; *GOS* galacto-oligosaccharides; *MFGM* milk fat globule membrane

In a neonatal animal model, dietary bLF has been suggested to promote intestinal maturation such as the development of crypt-villus structures and mucosal barrier, and increased enzyme activity (Yang et al. 2014; Hu et al. 2019). Intestinal development is important for the digestion and absorption of nutrients; therefore, bLF might have promoted body growth by improving the absorption of nutrients. Furthermore, bLF has been reported to suppress fat accumulation and promote lipolysis (Ono et al. 2013). bLF may facilitate efficient utilization of milk fat as an energy source. However, the intake of solid food or subtle differences in the amount of total protein in the test food can also affect the infant’s body growth. To evaluate the growth-promoting effect of bLF, further studies are needed under strict control of the test food composition and considering the effects of alternative nutrient sources such as solid food.

### Cerebral development and fecal microbiome

Several animal studies have suggested the potential involvement of bLF in neuroprotection, neurodevelopment, and learning. bLF administration through lactation showed a protective effect on impaired cerebral development in intrauterine growth restricted rats (Somm et al. 2014) and neuroprotective effect against hypoxia/ischemia-induced and LPS-induced brain injury (van de Looij et al. 2014; Ginet et al. 2016). bLF has also been reported to promote early cognitive function and learning in neonatal piglets (Chen et al. 2015b). On the other hand, there are limited clinical reports on the effect of bLF or bLF with other components on cerebral development (Tables 7, 8). One of them reported that bLF improved sleep quality (Miyakawa et al. 2020), but it was a secondary analysis of the study. Another reported improved neurodevelopment when bLF was administered in combination with MFGM (Li et al. 2019), but MFGM itself was also reported to have neurodevelopmental effects (Timby et al. 2014) and the net contribution of bLF did not evaluated. Due to above limitations, further clinical evidence is required.

**Table 7:** Clinical studies on the effect of bLF on cerebral development and fecal microbiome in children

Refs., study design	Population, country	bLF intake, form	Control	Intervention period	Results
Miyakawa et al. (2020) RDBPCT	109 healthy children, 12–32 months (Japan)	48 mg, formula	bLF-free formula	13 weeks	Improvement in sleep quality (morning symptoms)
Balmer et al. (1989) -	58 infants (England)	2.8 g/L ± iron, formula	bLF-free formula	14 days	Slight effect on the fecal microbiome
Roberts et al. (1992) -	51 healthy, formula-fed, term infants, at birth (Italy)	0.1 g/L, 1.0 g/L, formula	bLF-free formula	150 days	Established 'Bifidus flora' after 90 days in infants fed 1.0 g/L of bLF

*RDBPCT* randomized, double-blind, placebo-controlled trial

**Table 8:** Clinical studies on the effect of bLF with other components on cerebral development and fecal microbiome in children

Refs., study design	Population, country	bLF intake, form	Control	Intervention period	Results
Li et al. (2019) RDBPCT	451 healthy, formula-fed, term infants, 10–14 days (China)	0.6 g/L, + MFGM, formula	bLF-free, MFGM-free formula	~ 365 days of age	Higher Bayley-III scores in cognitive, language, and motor development on day 365. Partially higher scores related to neurodevelopment investigated using ASQ, CDI, TTS, and single object free play
Johnston et al. (2015) RDBPCT	480 healthy, formula-fed, term infants, 12–16 days (USA)	0.6 g/L, 1.0 g/L + PDX /GOS, formula	bLF-free, PDX-free, GOS-free formula	~ 365 days of age	Softer stool consistency in the bLF 0.6 and bLF 1.0 groups
Chichlowski et al. (2021) RDBPCT	451 healthy, formula-fed, term infants, 10–14 days (China)	0.6 g/L, + MFGM, formula	bLF-free, MFGM-free formula	~ 365 days of age	Subtle change in the fecal microbiome (*Bacteroides uniformis* and *Bacteroides plebeius* were abundant) at 4 months

*RDBPCT* randomized, double-blind, placebo-controlled trial; *MFGM* milk fat globule membrane; *Bayley-III* Bayley Scales of Infant Development, 3rd edition; *ASQ* Ages & Stages Questionnaire; *CDI* MacArthur-Bates Communicative Development Inventories; *TTS* Carey Toddler Temperament Scale; *PDX* polydextrose; *GOS* galacto-oligosaccharides

Differences in the fecal microbiome of breastfed infants and formula-fed infants have been indicated in previous studies (Yoshioka et al. 1983; Balmer and Wharton 1989), and the association of the fecal microbiome with immune function and cerebral development has also been discussed. In vitro studies have reported that bLF exhibits antibacterial activity against a wide range of pathogenic bacteria (Jenssen and Hancock 2009), whereas bLF and its digestive peptide show bifidogenic activity, particularly against infant-representative species (Oda et al. 2013). Increased secretion of HBD-2, cathelicidin, sIgA, and butyrate by bLF may also contribute to the regulation of the microbiome (Chen et al. 2021). However, no consistent effect of bLF or bLF with other components on the fecal microbiome was observed (Tables 7, 8). Compared with other components of breast milk such as human milk oligosaccharides and sIgA, the effect of LF on the fecal microbiome may be limited.

## Conclusions

bLF may contribute to the child health, particularly by suppressing or alleviating gastrointestinal and respiratory symptoms and improving the iron status of children with anemia or those at a high risk of anemia. The dose of bLF varies depending on the expected effect and target age, ranging from 35.8 to 1000 mg per day, and it may not necessarily have to be as high as that found in human breast milk. Subject characteristics, field conditions and quality of bLF may have led to inconsistent results. In addition, some beneficial effects of bLF were observed in the secondary analysis of the study. Further clinical evidence as the primary endpoint is needed to accurately describe the beneficial effects of bLF on the child health.

## Data Availability

The data presented in this review are openly available on PubMed, Google Scholar, and the journal website.
